# The Role of ER Stress in Bilirubin Neurotoxicity: A Complex Molecular Network

**DOI:** 10.3390/antiox15040451

**Published:** 2026-04-03

**Authors:** Mohammed Qaisiya, Claudio Tiribelli, Cristina Bellarosa

**Affiliations:** 1 Innovative Models Unit, Fondazione Italiana Fegato (Italian Liver Foundation), 34149 Trieste, Italy; qaisiyam@hebron.edu (M.Q.); ctliver@fegato.it (C.T.); 2 Department of Medical Laboratory Science, Hebron University, Hebron P785, Palestine

**Keywords:** unconjugated bilirubin, endoplasmic reticulum stress, calcium homeostasis, neuroinflammation, neurotoxicity

## Abstract

Although the molecular pathogenesis of bilirubin-induced neuronal cell injury is not completely understood, certain recurrent themes resonate in the literature on this topic and include the generally untoward effects of high unconjugated bilirubin (UCB) concentrations on membranes (plasma, mitochondrial, and endoplasmic reticulum (ER)), cellular bioenergetics, and intracellular calcium homeostasis. Only in the last decade, ER was discovered as an early target of bilirubin neurotoxicity. We will review the main features of bilirubin neurotoxicity from the point of view of ER and bilirubin-induced ER stress. Neuronal excitotoxicity, mitochondrial energy failure, and increased intracellular calcium concentration are three phenomena linked spatially and temporally in the pathogenesis of bilirubin-induced neurotoxicity. ER, being the main intracellular calcium storage organelle, is involved in the increase in the universal second messenger, calcium. This event leads to the activation of proteolytic enzymes, apoptotic pathways, and necrosis, the occurrence of which is likely a function of the degree and duration of bilirubin exposure.

## 1. Introduction

Unconjugated bilirubin (UCB) is the end product of heme catabolism, that exhibits cytoprotective and cytotoxic properties. At physiological concentrations, UCB functions as a potent endogenous direct antioxidant; however, when present at elevated levels—especially in cases of sever neonatal hyperbilirubinemia—it becomes neurotoxic [1]. Prolonged and severe hyperbilirubinemia is marked by elevated concentrations of UCB, which promote its accumulation [2] within the CNS [3], ultimately leading to a severe form of chronic bilirubin encephalopathy known as kernicterus. Kernicterus is associated with irreversible neurological sequelae, including motor abnormalities, auditory deficits, and learning impairments [4,5,6]. These central nervous system (CNS) manifestations reflect the regional distribution of bilirubin-induced neuropathology, which primarily affects the globus pallidus, subthalamic nucleus, brainstem nuclei, hippocampal CA2 neurons, and cerebellar Purkinje cells [7,8,9].

Among the emerging mechanisms, endoplasmic reticulum (ER) stress has gained attention in the last decade as an early and pivotal event of UCB-induced neuronal injury [10,11,12]. The ER represents an integrated molecular web that plays a central role in protein synthesis, calcium homeostasis, and glucose and lipid metabolism [13]. Perturbation of ER homeostasis leads to activation of the unfolded protein response (UPR), an orchestrated signaling that involves the activation of three main stress sensors (PERK, IRE1, and ATF6) [14]. While transient UPR activation promotes adaptive response and cellular survival, sustained or prolonged ER-stress shifts signaling toward pro-apoptotic pathways, notably via CHOP induction, inflammatory cascade, and calcium signaling [15]. Importantly, ER stress functions as a central node rather than an isolated event. ER has a specialized environment that makes it very sensitive to oxidative stress [16]. Simultaneously, disruption of ER calcium homeostasis triggers cytosolic calcium overload, activation of calpain and caspase pathways [17], and mitochondrial impairment [18]. Moreover, ER-stress sensors interface with inflammatory mediators such as NF-κB and JNK, triggering neuroinflammation [19].

Several studies demonstrated the important role of oxidative stress, calcium dysregulation, mitochondrial dysfunction, and inflammatory signaling in UCB-induced neurotoxicity [20]. However, the complex molecular mechanisms of UCB-induced neurotoxicity that lie on ER stress need further investigations. This review will first recapitulate the main known mechanisms of UCB neurotoxicity, then detail the molecular basis of ER stress, and focus on demonstrating how ER stress acts as a central hub interacting with pathways such as oxidative stress, calcium homeostasis, mitochondrial function, inflammation, and autophagy, ultimately determining neuronal fate. By framing ER stress as a systems-level regulator rather than a single linear pathway, we propose a conceptual model in which neuronal fate is determined by the dynamic balance between pro-survival and pro-apoptotic signaling. We hope this integrative perspective will provide new insights for future therapeutic strategies.

## 2. Molecular Mechanisms and Cellular Target of UCB-Induced Neurotoxicity

**Figure 1:**
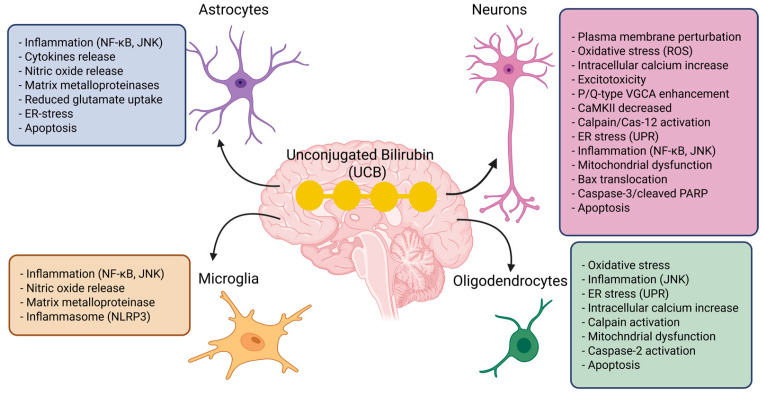
UCB exerts toxic effects on neurons, astrocytes, microglia, and oligodendrocytes. In neurons, UCB disrupts the plasma membrane, induces oxidative stress (ROS), increases intracellular calcium, promotes excitotoxicity, enhances P/Q-type voltage-gated calcium channels, decreases CaMKII, activates calpain and caspase-12, triggers endoplasmic reticulum stress (UPR), stimulates inflammatory pathways such as NF-κB and JNK, impairs mitochondrial function, causes Bax translocation, caspase-3 activation, and PARP cleavage, ultimately leading to apoptosis. In astrocytes, UCB promotes inflammation through NF-κB and JNK, with release of cytokines and nitric oxide, increased matrix metalloproteinases, reduced glutamate uptake, ER stress, and, under severe conditions, apoptosis. Microglia respond with rapid inflammatory activation, characterized by NF-κB/JNK signaling, release of nitric oxide, increased matrix metalloproteinases, and activation of the NLRP3 inflammasome, thereby amplifying neuroinflammation. Oligodendrocytes are also highly vulnerable: UCB induces oxidative stress, JNK-mediated inflammation, ER stress (UPR), intracellular calcium increase, calpain activation, mitochondrial dysfunction, caspase-2 activation, and finally apoptosis. Created in BioRender. Models, I. (2026) https://BioRender.com/gzshrzf (accessed on 25 March 2026).

UCB is highly lipophilic and binds strongly to cell membranes, particularly myelin-rich membranes, making neurons the main targets of bilirubin toxicity. Non-neuronal cells in the CNS are also affected by UCB and may modulate bilirubin-induced neurotoxicity [21]. Figure 1 shows how UCB affect neurons, astrocytes, microglia, and oligodendrocytes. In vitro, neuronal exposure to UCB leads to reduced dendritic and axonal arborization, impaired neurite growth and branching, decreased proliferation, and increased apoptosis [22,23]. In neuroblastoma cells, bilirubin delays S-phase progression and induces cell-cycle arrest [22], a mechanism that may contribute to the cerebellar hypoplasia observed in murine models of kernicterus Bilirubin, and also causes major biochemical disturbances, including protein oxidation, lipid peroxidation, glutathione depletion, increased lactate dehydrogenase release, and nitric oxide production through NMDA receptor-mediated activation of neuronal nitric oxide synthase [24]. These events are closely associated with oxidative stress and mitochondrial dysfunction, which appear to be central mechanisms of neuronal injury. Astrocytes respond to toxic bilirubin levels by releasing inflammatory mediators such as IL-1β, TNF-α, and IL-6, as well as glutamate, and may ultimately undergo apoptosis, although they are generally less vulnerable than neurons [25]. Microglia, directly activated by UCB, acquire a phagocytic phenotype, secrete pro-inflammatory cytokines, and increase matrix metalloproteinase activity, supporting the idea that bilirubin encephalopathy involves a strong neuroinflammatory component [26]. Oligodendrocytes are likewise susceptible, showing mitochondrial impairment, increased reactive oxygen species, and caspase-3-mediated apoptosis; this may contribute to altered myelin production and axonal dysfunction in brain regions typically damaged in kernicterus [27].

Although neurons are the most sensitive cell type, non-neuronal cells also actively contribute to the progression of injury. Overall, the neurotoxicity of unconjugated bilirubin results from the combined effects of oxidative stress, calcium imbalance, inflammation, and altered glutamatergic transmission, which will be analyzed in detail below.

### 2.1. Oxidative Stress: A Central and Early Event

Oxidative stress represents a pivotal mechanism in bilirubin neurotoxicity and occurs when reactive oxygen species (ROS) generation overwhelms endogenous antioxidant defenses. Bilirubin displays a well-recognized “Janus-like” behavior: at low intracellular concentrations it exerts antioxidant effects, whereas at higher levels it becomes pro-oxidant [20]. In vitro studies in hepatic, endothelial, renal, and neuronal cell lines demonstrated that bilirubin acts as an antioxidant at approximately 7 ng/mg protein intracellularly, while concentrations exceeding 25 ng/mg protein shift its activity toward pro-oxidant effects [28].

At toxic concentrations, bilirubin enhances ROS production, promotes lipid and protein oxidation, and disrupts glutathione metabolism [29]. Oxidative imbalance contributes to the activation of mitochondrial apoptotic pathways [30,31]. Furthermore, UCB-induced oxidative stress affects DNA integrity by promoting oxidative DNA damage. In primary culture of neurons, UCB-induced ROS has been directly linked to Tet1 (ten-eleven translocation 1) protein inhibition and increased DNA methylation [32]. Cells attempt to counteract oxidative stress by upregulating compensatory mechanisms, including enhanced glutathione synthesis [22], activation of the redox regulator APE1/Ref-1 (apurinic/apyrimidinic endonuclease 1/redox effector factor 1) [33], activation of the stress-response adaptive protein DJ-1 (Parkinson disease protein 7) [22], and the activation of Nrf2-dependent antioxidant gene programs [34]. However, when these adaptive responses are insufficient, oxidative damage becomes a major driver of neuronal dysfunction.

### 2.2. Membrane Perturbation and Calcium Dysregulation

An enduring hypothesis [7] proposes that neuronal injury begins at the membrane level, where bilirubin perturbs membrane structure and dynamics. Disruption of plasma, mitochondrial, and endoplasmic reticulum (ER) membranes contributes to intracellular calcium overload. The resulting elevation in the universal second messenger Ca^2+^ activates downstream cascades, including protease activation, mitochondrial dysfunction, apoptotic signaling, and necrosis [30,35].

In parallel, bilirubin induces ER stress [10,36], with gene signatures resembling thapsigargin exposure. ER calcium release leads to sustained calcium elevations—more pronounced in neurons than in glia—and activation of ER stress-, calpain-, and calcium-dependent apoptotic pathways [37]. One possible scenario is that ER-derived Ca^2+^ release can be transferred to mitochondria through ER–mitochondria contact sites (MAMs), promoting mitochondrial Ca^2+^ overload, bioenergetics failure, and amplification of apoptotic signaling. Autophagy is also activated as an early protective response [38]. Collectively, these findings identify calcium dysregulation and ER stress as central and potentially targetable mechanisms.

### 2.3. Inflammation and Glial Contribution

Beyond oxidative stress, inflammation represents the most extensively studied pathogenic mechanism of bilirubin toxicity. Neurons exposed to bilirubin activate pro-inflammatory and pro-apoptotic pathways—including IL8, NOS signaling, and caspase cascades—with exacerbated injury in the presence of TNFα and IL1β, and impaired neurite outgrowth and synaptogenesis [11,39,40]. Although neurons are particularly vulnerable, glial cells actively amplify injury. Microglia and astrocytes release cytokines, nitric oxide, and matrix metalloproteinases, thereby sustaining neuroinflammation [25,26,41]. Conversely, blood–brain barrier interfaces display limited cytokine modulation in vivo and may regulate immune cell infiltration.

### 2.4. Glutamatergic Dysfunction and Excitotoxicity

Glutamate-mediated excitotoxicity provides a mechanistic link between cellular injury and regional brain vulnerability. Bilirubin-stimulated glial cells release glutamate [42,43], while both neurons and astrocytes exhibit reduced glutamate uptake [44]. The consequent extracellular glutamate accumulation results in NMDA receptor overactivation, calcium influx, and excitotoxicity [45,46].

Transcriptomic alterations in glutamatergic synapse-related genes in susceptible brain regions [47] parallel neuroimaging findings in affected neonates [48]. Glutamatergic dysfunction and excitotoxicity have therefore been proposed as unifying mechanisms linking regional susceptibility to the clinical topography of bilirubin-induced brain injury [20].

## 3. Molecular Basis of ER-Stress

The endoplasmic reticulum (ER) has a specialized oxidized environment that allows proper protein folding, making the ER extremely sensitive to alteration in cellular redox states and calcium homeostasis [49]. In addition to its role in protein quality control, the ER is also considered as a “nutrient-sensing apparatus” through its ability to control energy, glucose, and lipid metabolism [50]. ER stress is triggered by the changing of cellular redox state, calcium disruption, or the accumulation of misfolded proteins, which collectively initiates a cascade of signaling pathways known as the unfolded protein response (UPR) [51]. The UPR involves three main membrane-associated proteins: PERK (PKR-like endoplasmic reticulum kinase of the eukaryotic initiation factor 2α), IRE1 (inositol-requiring enzyme 1), and ATF6 (activating transcription factor-6). Under basal conditions, these three proteins are kept inactive through their interaction with the chaperone BiP (or GRP78), which dissociates by the misfolded proteins under ER-stress conditions.

Upon activation, PERK phosphorylates eukaryotic initiation factor 2 (elF2α), leading to the attenuation of protein synthesis and selective upregulation of ATF4. ATF4 induces the upregulation of CHOP that contributes to cell-cycle arrest by decrease in cyclin D1. Activated IRE1 mediates the selective splicing of XBP1 mRNA, which results in a stable, active transcription factor. ATF6 translocates to the Golgi apparatus where it undergoes proteolytic cleavage, generating a cleaved-active transcription factor [52]. All three UPR branches induce transcription factors (e.g., ATF4, CHOP, sXBP1, and ATF6) that reprogram the cellular expression profile by upregulating genes involved in stress response, inflammation, antioxidants, and apoptosis [14,19,53].

## 4. ER-Stress as a Central Integrator of UCB Toxicity In Vitro and In Vivo

**Figure 2:**
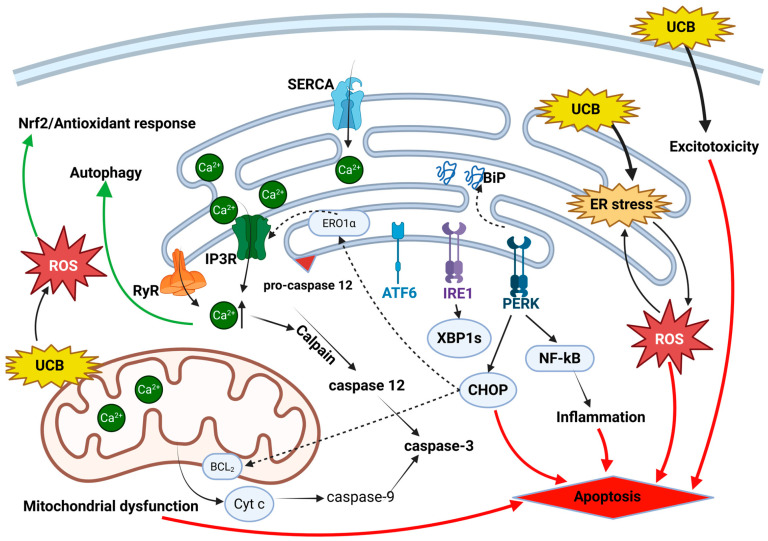
Molecular pathways dysregulated in bilirubin-induced neurotoxicity. Unconjugated bilirubin (UCB) triggers a coordinated pro-apoptotic signaling network integrating ER stress, calcium dysregulation, reactive oxygen species (ROS) generation, inflammation, excitotoxicity, and mitochondrial dysfunction. UCB disrupts cellular redox state and induces the activation of ER stress. The chaperone BiP dissociates from PERK and IRE1 and binds to the misfolded proteins, initiating the unfolded protein response. PERK phosphorylates eIF2α, with subsequent activation of the ATF4/CHOP axis and downstream inflammatory signal including NF-κB. IRE1 and ATF6 activate a transcriptional program that amplifies stress signals. UCB-induced ER stress perturbs calcium homeostasis, promoting Ca^2+^ release from the ER and activation of calpain and caspase-12, thereby further driving apoptotic signaling. Pro-survival pathways are concurrently activated to counteract UCB toxicity, including the Nrf2-mediated antioxidant response and autophagy. Cellular fate is determined by the intensity and duration of UCB exposure, reflecting the balance between adaptive stress responses and pro-apoptotic signaling. Pro-apoptotic signaling flows are indicated in red, while pro-survival signaling flows in green. Created in BioRender. Models, I. (2026) https://BioRender.com/gzshrzf (accessed on 25 March 2026).

Cells must maintain bilirubin at very low, non-toxic intracellular levels through multiple protective mechanisms. As shown in Figure 2, after entering the cell, unconjugated bilirubin (UCB), due to its poor aqueous solubility, binds to intracellular ligandins and is shuttled toward the endoplasmic reticulum (ER), which acts as a “sink” for excess UCB [54]. Direct accumulation of UCB within the ER may represent a primary trigger of ER stress, as UCB can interact with hydrophobic regions of proteins, thereby interfering with proper protein folding and ER homeostasis. This proposed mechanism largely depends on the capacity of the cell, particularly the ER, to detoxify UCB.

Unlike hepatocytes, most cell types lack an efficient conjugation system and therefore rely on alternative pathways for UCB elimination [55]. One such pathway involves UCB oxidation by cytochrome P450 enzymes, which are abundant in the ER [56,57,58]. Cytochrome P450 enzymes are expressed in a tissue- and cell type-specific manner and are localized to different subcellular compartments, reflecting their diverse physiological and pathological roles [59]. Under conditions of elevated UCB, the activity or expression levels of specific cytochrome P450 isoforms may determine the susceptibility of individual cell types to UCB-induced ER stress. Further studies are needed to clarify how UCB affects ER protein-folding processes, and to determine whether variability in ER-stress sensitivity is directly related to differences in cytochrome P450 expression and activity across cell types.

ER-stress induction following UCB exposure has been reported in several in vitro models. Neuronal cell lines including SH-SY5Y and GI-ME-N show upregulation of ATF4, CHOP, ATF6, and BiP after 4 h of exposure [11,36]. Similarly, a primary culture of mouse hippocampal neurons exhibits increased protein content of CHOP, cleaved-ATF6, and BiP upon exposure to UCB for 24 h [37]. In rat oligodendrocyte precursor cultures, UCB exposure induces BiP at 1 h followed by an-upregulation of IRE-1α and ATF-6 at 2 h [60]. Microarray analysis performed on Hepa 1c1c7 cells exposed to UCB revealed a phosphorylation of PERK and elF2α after 1 h and an upregulation of CHOP, ATF3, and BiP [61]. Recent in vivo studies further support these findings. In the UGT1A1 knockout mouse model, it showed that the exposure of a developing cerebellum to sustained UCB levels induces the activation of ER-stress markers (ATF3 and CHOP) at the early stages of disease onset [12]. Moreover, activation of UPR-related genes, such as ATF3, CHOP, DDIT4, XBP1, and Fos has been reported in a mouse model of acute UCB-induced neurotoxicity [10]. Collectively, these studies support the concept that ER stress represents an early and central event of UCB toxicity.

### 4.1. Crosstalk Between UCB-Induced ER-Stress and Oxidative Stress

Accumulating evidence indicates that ER stress and oxidative stress are closely interconnected processes [49]. Unlike other organelles, the ER possesses a unique oxidizing environment that supports proper protein folding and disulfide bond formation. This characteristic makes the ER highly sensitive to changes in the cellular redox state. Under physiological conditions, the ER generates relatively high levels of reactive oxygen species (ROS) due to the presence of numerous oxidoreductase enzymes, such as ERO1, NOX4, and cytochrome P450, which participate in electron transfer reactions and ROS production [62]. When the activity of these enzymes becomes dysregulated, or when cells are exposed to conditions that alter the intracellular redox balance (e.g., changes in the GSH/GSSG ratio), misfolded proteins accumulate and the unfolded protein response (UPR) is activated as an adaptive mechanism [49]. ROS also modulates the activity of stress-responsive kinases, including PKC, PI3K, MEK/ERK1, and p38α, which contribute to cell survival or death. Although multiple kinases can be activated by ROS, the specific signaling response depends on the cell type and the nature of the inducing stimulus [63].

Several studies have shown that oxidative stress and depletion of intracellular glutathione (GSH) are hallmarks of UCB-induced neurotoxicity [12,22,29,33,64]. In response, cells activate the antioxidant mechanisms, where the Nrf2/HO-1 pathway, a master regulator of the antioxidant response machinery has been getting significant attention [34]. In addition, under ER-stress conditions, Nrf2 can be directly activated by PERK, contributing to adaptive responses [53].

Although numerous studies demonstrate increased oxidative stress following UCB exposure, it remains unclear whether oxidative stress is a primary trigger of ER stress or a secondary event downstream of ER dysfunction. Our previous studies in SH-SY5Y neuronal cells showed that the induction of antioxidant genes occurs later than the activation of ER stress-related genes [34], suggesting that oxidative stress is a secondary event in UCB toxicity. Consistent with this observation, treatment of SH-SY5Y cells with the antioxidant N-acetylcysteine (NAC) did not affect the expression of ER-stress markers, whereas treatment with the ER-stress inhibitor 4-phenylbutyrate (4-PBA) reduced the expression of the Nrf2-dependant HO-1 mRNA gene, a marker of oxidative stress (unpublished data).

### 4.2. UCB-Induced ER-Stress and Calcium Disruption

Cells tightly control intracellular calcium (Ca^2+^) levels through an integrated network of channels and transport systems located in the plasma membrane, mitochondria, and endoplasmic reticulum (ER) [65]. The ER functions as a dynamic Ca^2+^ reservoir and maintains Ca^2+^ concentrations that are several orders of magnitude higher than those in the cytosol, a condition essential for proper protein folding and ER function. Calcium release from the ER is primarily mediated by inositol 1,4,5-trisphosphate receptors (IP_3_Rs) and ryanodine receptors (RyRs), which are activated by IP_3_ and cytosolic Ca^2+^, respectively. Excess cytosolic Ca^2+^ is actively transported back into the ER by the sarco-/endoplasmic reticulum Ca^2+^-ATPase (SERCA) pumps [66]. Multiple isoforms of IP_3_Rs and RyRs are expressed in the brain, where they play critical roles in maintaining neuronal survival; however, their dysregulation can also contribute to apoptotic cell death [67].

Alterations in intracellular Ca^2+^ homeostasis can act both as a trigger for ER stress and as a consequence of it. Depletion of ER Ca^2+^ stores activates the unfolded protein response (UPR), and when the stress is severe or prolonged, ER stress promotes cell death [65]. Several ER-stress inducers, such as thapsigargin and EGTA, cause profound ER Ca^2+^ depletion, whereas others, including tunicamycin and dithiothreitol (DTT), activate the UPR without significantly reducing ER Ca^2+^ levels. These observations indicate that ER Ca^2+^ depletion is not an obligatory requirement for UPR activation [68].

One mechanism contributing to ER Ca^2+^ depletion involves the CHOP induction. During ER stress, CHOP upregulates ER oxidase 1α (ERO1α), which enhances IP_3_R activity and promotes Ca^2+^ release from the ER [69]. The resulting elevation in cytosolic Ca^2+^ activates the calpain/caspase-12 pathway, contributing to ER stress-mediated apoptosis [17].

An increase in intracellular Ca^2+^ levels following exposure to UCB has been reported in several in vitro studies, including in SH-SY5Y cells [38] and primary cultures of neurons [37,70]. Additionally, an ex vivo model of hippocampal slices treated with UCB (acute model) or derived from the Gunn rat (endogenous model) showed a strong Ca^2+^ oscilation in pyramidal cells that involved both membrane Ca^2+^ influx and intracellular sources [71]. Brain slices prepared from the ventral cochlear nucleus show that UCB treatment increases calcium entry into developing auditory neurons by enhancing P/Q-type voltage-gated calcium channel activity, potentially contributing to neurotoxicity [72]. Consistent with these observations, an in vivo study demonstrated that 24 h after UCB administration, intracellular free Ca^2+^ concentrations were significantly elevated, accompanied by increased caspase-3 activation and neuronal apoptosis [73]. Direct evidence for the involvement of ER stress in UCB-induced Ca^2+^ dysregulation has been demonstrated in mouse hippocampal neurons. UCB exposure induced large intracellular Ca^2+^ oscillations that occurred independently of action potential activity. Pharmacological inhibition of ER Ca^2+^ reuptake further confirmed the ER as a major source of Ca^2+^ release, indicating that UCB disrupts ER Ca^2+^ homeostasis and depletes ER Ca^2+^ stores. This ER Ca^2+^ imbalance was associated with the activation of the calpain/caspase-12 pathway [37]. Furthermore, UCB interferes with neuronal intracellular calcium homeostasis by altering the function and expression of calcium/calmodullin-dependent kinase II (CaMKII). UCB inhibits CaMKII and causes selective decreases in the protein content in the susceptible area, increasing the neuronal intracellular calcium [74]. While UCB acts as an inhibitor of CaMKII, it has also been shown to involve calmodulin (CaM) in augmenting VGCC (voltage-gated calcium channel) currents, contributing to a “calcium load” that triggers toxic effects in developing neurons [72].However, further studies are needed to determine whether Ca^2+^ overload results primarily from enhanced extracellular Ca^2+^ influx, increased release from intracellular stores, or a combination of both.

### 4.3. UCB-Induced ER Stress Shows Possible Link to Mitochondrial Dysfunction

During chronic ER stress, the major contributor of cell death results from crosstalk between the ER and mitochondria occurring at multiple levels [75]. First, ER stress promotes calcium release, which can trigger mitochondrial permeability transition pore (mPTP) opening, ATP depletion, increased mitochondrial ROS production, caspases activation and apoptosis [76]. Second, ER stress-induced CHOP upregulates ERO1α activity, thereby enhancing ROS generation and promoting cell death [69]. Third, CHOP downregulates anti-apoptotic BCL-2 proteins while upregulating pro-apoptotic members (PUMA, BIM, and NOXA), leading to increased oxidative stress, mitochondrial dysfunction, and apoptosis [77]. Forth, ER stress-associated ROS promotes physical interactions between the ER and mitochondria through the formation of mitochondria-associated membranes (MAMs), further amplifying stress and cell death signaling [78].

Direct evidence further supports mitochondrial injury following UCB exposure. UCB inhibits cytochrome c oxidase activity in isolated mitochondria [79,80] and immature cortical neurons [81]. Hepa 1c1c7 cells treated with UCB exhibits mitochondrial membrane depolarization, cytochrome c release, apoptosome formation and caspase-9/-3 activation [31]. UCB also induces mitochondrial depolarization and Bax translocation, mediating the mitochondrial pathway of apoptosis in primary rat neurons [40]. UCB exerts significant, concentration- and cell type-dependent effects on cellular metabolism by inhibiting respiration, ATP production, and disrupting the TCA cycle, thereby promoting ROS production and metabolic dysfunction [82]. In neurons which are highly energy-demanding and rely predominantly on oxidative phosphorylation (OXPHOS), this energy deficit directly impairs cellular function and promotes apoptosis. In glial cells, mitochondrial inhibition can trigger a compensatory shift toward the glycolytic pathway that influences glial activation states, potentially promoting pro-inflammatory signaling [83].

Oligodendrocyte precursor cells exposed to UCB undergo apoptosis, showing early signals of ER-stress markers, loss of mitochondrial membrane potential, and caspase-2 activation. The activation of caspase-2 is often considered an apical initiator that links ER stress to mitochondrial apoptosis [60]. While mechanistic links between ER–mitochondrial crosstalk in UCB-induced neurotoxicity have been proposed, direct evidence regarding the temporal sequence and cell-specific outcomes is lacking, highlighting the need for further research in this area.

### 4.4. UCB-Induced ER Stress Triggers Neuroinflammation

Persistent ER stress is known to elicit inflammatory responses and cell injury through multiple mechanisms [84]. The three major UPR branches contribute to inflammation, primarily through activation of the JNK and NF-κB signaling pathways [50,84]. Activated IRE1α recruits tumor necrosis factor receptor-associated factor 2 (TRAF2), and this complex interacts with and activates JNK and IκB kinase (IKK) while the XBP1s promote the production of pro-inflammatory cytokines such as IL-6 and IL-8 [85]. PERK-mediated translational attenuation leads to the release of NF-κB from its inhibitor IκB, thereby inducing the expression of multiple genes involved in inflammatory pathways [86]. In addition, the PERK/ATF4/CHOP axis contributes to the induction of inflammatory cytokines [87]. The induction of inflammation by specific arm(s) of UPR is probably dependent on cell type and inducer. Besides the direct role of ER stress in mediating inflammation, other factors—including reactive oxygen species (ROS) and nitric oxide (NO) production, disturbed Ca^2+^ homeostasis, and mitochondrial dysfunction—act synergistically to amplify the inflammatory response [85].

In vitro studies using primary neuronal cultures have demonstrated activation of NF-κB and increased expression of TNF-α, IL-1β, and IL-6 following UCB exposure [88,89]. Primary rat cortical microglia and astrocytes also exhibit NF-κB and JNK activation, leading to the upregulation of TNF-α, IL-1β, and IL-6 in response to UCB [25]. In our previous study, we demonstrated a link between ER stress and inflammation. UCB-induced IL-8 expression via NF-κB in SH-SY5Y cells was significantly reduced by the ER-stress inhibitor (4-PBA) and PERK silencing, confirming the contribution of ER stress to neuroinflammation [11]. In vivo, cerebellar mRNA levels of NF-κB, IL-18, and TNF-α are increased in UGT1^−/−^ mice compared with controls [12]. Yueh et al. further demonstrated that the UGT1^−/−^ mouse model exhibits activation of NF-κB and JNK and increased expression of TNF-α, IL-1β, and IL-6, creating a pro-inflammatory CNS environment via TLR2 signaling [90]. UCB induces inflammasome (NLR family pyrin domain containing three (NLRP3)) in N9 microglial cells and microglia of neonatal mice [91]. Emerging evidence indicates that Toll-like receptor (TLR) signaling and ER-stress pathways interact synergistically to amplify pro-inflammatory responses. TRAF-related proteins, such as TRAF2, can be recruited by both TLR2/4 and IRE1α, providing a potential mechanistic link between TLR signaling, ER stress, and NF-κB/JNK activation [85,92,93]. However, the contribution of ER stress to UCB-induced neuroinflammation still requires further investigation.

### 4.5. UCB-Induced ER Stress Activates Autophagy

Autophagy can be potently activated by ER stress [94]. ER stress induces autophagy as an adaptive mechanism to eliminate misfolded proteins and damaged organelles during the recovery phase following cellular stress. Ogata et al. demonstrated that ER stress-induced autophagy is regulated through IRE1α/TRAF2/JNK activation [95]. Margariti et al. reported that XBP1 mRNA splicing promotes autophagy through transcriptional activation of Beclin-1 [96]. Activation of the PERK arm of the unfolded protein response has also been shown to induce autophagy through ATF4-mediated transcriptional regulation of autophagy-related (ATG) genes [97].

Only a limited number of studies have investigated the role of UCB in autophagy induction. Upregulation of autophagy-related genes in human neuroblastoma SH-SY5Y cells following UCB exposure has been previously reported [36]. High levels of UCB also induce autophagy as a late response, as evidenced by the conversion of LC3-I to LC3-II in human brain microvascular endothelial cells [98]. Autophagy activation was confirmed in the cerebellum of Ugt1^−/−^ mice as a delayed event triggered by UCB exposure [12]. A link between ER stress and autophagy activation was demonstrated in SH-SY5Y cells exposed to UCB. Pharmacological inhibition of ER stress significantly reduced UCB-induced autophagy, through ER-stress/mTOR/Ca^2+^ signaling. Importantly, our findings revealed that autophagy activation in neuronal cells plays a protective role against UCB toxicity by reducing HO-1 expression (oxidative stress marker), inflammation, and CHOP expression [38]. Autophagy facilitates the removal of damaged mitochondria (mitophagy), other oxidant-generating organelles, and oxidized macromolecules, thereby limiting ROS accumulation in neurons and glial cells [99]. Autophagy suppresses the activation of pro-inflammatory cytokines and their receptors, including NF-κB signaling, and regulates inflammasome by mediating the degradation of NLRP3 [100]. Furthermore, autophagy enhances the clearance of the misfolded proteins and damaged ER components, reducing ER-stress/CHOP apoptotic signaling [101]. Further studies are needed to determine whether pre-activation of autophagy in vivo could mitigate UCB-induced neurotoxicity in the central nervous system.

Another important degradation pathway downstream of ER stress, is the ER-associated degradation system (ERAD), which is a key component of protein quality control that guarantees ER homeostasis by facilitating protein refolding and degradation, thereby protecting cells from misfolded protein-induced toxicity [102]. ERAD-related genes are regulated by PERK/ATF4, IRE1/XBP1s, and ATF6, and function to eliminate the misfolded proteins arised from ER stress through retrotranslocation activity across the ER membrane into the cytosol, where ubiquitin-conjugating enzymes target them for 26S proteosomal degradation [103]. Notably, impairment of the ERAD system is common among the broad spectrum of neuropathology disorders [104]. To our knowledge, one study has demonstrated the induction of several ERAD genes by UCB, including: HERP (Homocysteine-induced ER Protein), EDEM1 (ER Degradation Enhancer Mannosidase alpha-like 1), SEL1L (SEL1L sdaptor subunit of SYVN1 ubiquitin ligase), and DERL1 (Derlin membrane-like domain family member 2) [36]. Future studies using additional in vitro and in vivo models are required to further elucidate the mechanism of the UCB-induced ERAD system and its relation to neurotoxicity in selective brain regions.

### 4.6. ER Stress, a Molecular Switch of Adaptive Versus Apoptotic Responses to UCB

ER stress-activated pathways are complex, and when cells fail to restore homeostasis, a rapid transition toward apoptotic signaling occurs [105]. The most extensively studied mechanism of ER stress-induced cell death involves CHOP induction. CHOP promotes apoptosis through multiple mechanisms: it downregulates the anti-apoptotic protein Bcl-2, upregulates pro-apoptotic genes (e.g., PUMA, NOXA, BAX, and BAK), induces ERO1α, thereby increasing oxidative stress, and stimulates GADD34 expression, which restores protein translation and further exacerbates ER stress [77]. Another key mediator of ER stress-induced apoptosis is intracellular Ca^2+^ signaling. ER calcium release activates calpain (Ca^2+^-dependent proteases), which subsequently trigger the caspase-12/caspase-9 pathway. In addition, Ca^2+^ released from the ER is taken up by mitochondria, leading to mitochondrial Ca^2+^ overload, cytochrome c release, and activation of the caspase cascade [76]. The ER and mitochondria are structurally and functionally connected through mitochondrial-associated membranes (MAMs), which contain protein complexes such as IP3R, GRP75, MFN2, and VDAC [75]. Under ER stress conditions, enhanced ER–mitochondria coupling can lead to sustained. During chronic ER stress, PERK has been shown to localize at MAM sites, facilitating ER–mitochondria tethering and promoting rapid transfer of reactive oxygen species (ROS) from the ER to mitochondria. This process contributes to cytochrome c release and may also promote the redistribution of Bcl-2 family proteins to mitochondria [78]. The IRE1α branch also contributes to apoptosis during prolonged ER stress through activation of the TRAF2–ASK1–JNK pathway, resulting in Bcl-2 inhibition and caspase-12 activation [14].

We previously demonstrated that ER stress plays a central role in UCB-induced neuronal apoptosis, with increased CHOP expression and caspase-3 activation. CHOP KO reduces UCB-induced neurotoxicity while pharmacological reduction in ER stress by 4-PBA significantly attenuated UCB-induced neuronal cell death [11]. CHOP induction has also been reported in Hepa 1c1c7 cells exposed to UCB, accompanied by caspase-12 activation [31], in the cerebellum of UGT1A1 knockout mice [12]. Similarly, oligodendrocyte precursor cells exposed to UCB undergo apoptosis associated with ER stress, calpain activation, and JNK signaling [60].

As shown in Figure 2, one possible explanation for cell type-specific susceptibility to UCB is the differential ability to maintain adaptive ER-stress responses. More resistant cells, such as astrocytes, may remain in a pro-survival phase through activation of Nrf2 and autophagy, whereas vulnerable cells, such as neurons, may undergo unresolved ER stress, leading to apoptosis driven by ROS accumulation, CHOP induction, Ca^2+^ dysregulation, and neuroinflammation.

## 5. Conclusions

Significant progress has been made in recent years toward understanding the mechanisms underlying UCB-induced neurotoxicity. Emerging evidence in the last decade indicates that the endoplasmic reticulum (ER) stress pathway represents a central integrative mechanism linking oxidative stress, inflammation, autophagy, Ca^2+^ dysregulation, mitochondrial dysfunction, and apoptosis. However, further in vivo studies are needed to clarify how ER stress modulates these interconnected signaling networks and how disturbances within this pathway determine the balance between cell survival and cell death.

Understanding the pathological conditions under which these pathways are activated, and the mechanisms through which they ultimately influence cell fate, will facilitate the identification of key molecular targets and support the development of targeted therapeutic strategies aimed at preventing or reducing UCB-induced neurotoxicity.

## Data Availability

No new data were created or analyzed in this study. Data sharing is not applicable to this article.

## Abbreviations

The following abbreviations are used in this manuscript:

UCB	Unconjugated bilirubin
ATF4	Activating transcription factor 4
ATF6	Activating transcription factor 6
CHOP	C/EBP homologous protein
BiP	Binding immunoglobulin protein (GRP78)
eIF2α	Eukaryotic initiation factor 2 alpha
GRP78	Glucose-regulated protein 78
IRE1α	Inositol-requiring enzyme 1 alpha
PERK	PKR-like endoplasmic reticulum kinase
UPR	Unfolded protein response
sXBP1	Spliced X-box binding protein 1
DDIT4	DNA damage-inducible transcript 4
ERO1α	Endoplasmic reticulum oxidase 1 alpha
HO-1	Heme oxygenase 1
ATG	Autophagy-related gene
Bak	Bcl-2 homologous antagonist/killer
Bax	Bcl-2–associated X protein
Bcl-2	B-cell lymphoma 2
ASK1	Apoptosis signal–regulating kinase 1
GADD34	Growth arrest and DNA damage-inducible protein 34
NF-κB	Nuclear factor kappa B
IKK	IκB kinase
IL	Interleukin
IP_3_R	Inositol 1,4,5-trisphosphate receptor
JNK	c-Jun N-terminal kinase
LC3	Microtubule-associated protein 1 light chain 3
MAM	Mitochondria-associated membrane
mPTP	Mitochondrial permeability transition pore
mTOR	Mechanistic target of rapamycin
NAC	N-acetylcysteine
NO	Nitric oxide
NOXA	Phorbol-12-myristate-13-acetate-induced protein 1
Nrf2	Nuclear factor erythroid 2–related factor 2
PUMA	p53 upregulated modulator of apoptosis
ROS	Reactive oxygen species
RyR	Ryanodine receptor
SERCA	Sarco/endoplasmic reticulum Ca^2+^-ATPase
TLR	Toll-like receptor
TRAF2	TNF receptor-associated factor 2
UGT1A1	UDP-glucuronosyltransferase 1A1
CaMKII	Calcium/calmodullin-dependent kinase II
NLRP3	NLR family pyrin domain containing 3
ERAD	ER-associated degradation
HERP	Homocysteine-induced ER Protein
EDEM1	ER Degradation Enhancer Mannosidase alpha-like 1
SEL1L	SEL1L sdaptor subunit of SYVN1 ubiquitin ligase
DERL1	Derlin membrane-like domain family member 2
Tet1	Ten-eleven translocation 1
APE1/Ref-1	Apurinic/apyrimidinic endonuclease 1/redox effector factor 1
DJ-1	Parkinson disease protein 7
